# Genetic and immune microenvironment characterization of HER2‐positive gastric cancer: Their association with response to trastuzumab‐based treatment

**DOI:** 10.1002/cam4.5769

**Published:** 2023-03-14

**Authors:** Hyun Jung Kwon, Yujun Park, Soo Kyung Nam, Enoch Kang, Ka‐Kyung Kim, Inhae Jeong, Yoonjin Kwak, Jeesun Yoon, Tae‐Yong Kim, Keun‐Wook Lee, Do‐Youn Oh, Seock‐Ah Im, Seong‐Ho Kong, Do Joong Park, Hyuk‐Joon Lee, Hyung‐Ho Kim, Han‐Kwang Yang, Hye Seung Lee

**Affiliations:** ^1^ Department of Pathology Seoul National University Bundang Hospital Seongnam Republic of Korea; ^2^ Department of Pathology Seoul National University Hospital, Seoul National University College of Medicine Seoul Republic of Korea; ^3^ Cancer Research Institute Seoul National University Seoul Republic of Korea; ^4^ Integrated Major in Innovative Medical Science Seoul National University Graduate School Seoul Republic of Korea; ^5^ Seoul National University College of Medicine Seoul Republic of Korea; ^6^ Macrogen Inc. Seoul Republic of Korea; ^7^ Department of Internal Medicine Seoul National University Hospital, Seoul National University College of Medicine Seoul Republic of Korea; ^8^ Department of Internal Medicine Seoul National University Bundang Hospital, Seoul National University College of Medicine Seoul Republic of Korea; ^9^ Department of Surgery Seoul National University Hospital, Seoul National University College of Medicine Seoul Republic of Korea; ^10^ Department of Surgery Seoul National University Bundang Hospital, Seoul National University College of Medicine Seoul Republic of Korea

**Keywords:** cell cycle, HER2‐positive gastric cancer, NK cell, PD‐L1, trastuzumab

## Abstract

**Background:**

We aimed to determine the molecular and immune microenvironment characteristics of HER2‐positive gastric cancer (GC) related to the patient's response to first‐line trastuzumab‐based treatment.

**Methods:**

Eighty‐three cases of HER2‐positive advanced gastric adenocarcinoma patients treated with trastuzumab were enrolled. Targeted deep sequencing and transcriptome analysis were performed on selected 21 cases (exploration cohort) along with two post‐treatment samples. The results were compared between patients progressed before 6 months (Group 2) and others (Group 1), and were validated by FISH and immunohistochemistry in total cohort. Tumor‐infiltrating immune cells were evaluated using RNA sequencing data and multiplex immunohistochemistry. Progression‐free survival (PFS) analysis was performed.

**Results:**

Group 1 showed frequent amplification of G1/S cell cycle checkpoint‐related genes and upregulated KEGG pathways related to cell proliferation. In contrast, Group 2 had more frequent EGFR, HER3, and MET amplification and higher RNA expression in immune‐related KEGG pathways than Group 1. In total cohort, significant predictors of better PFS were cell cycle‐related including CCNE1 amplification, Cyclin A and PLK1 overexpression, and decreased Cyclin D3 and HER3 expression (*p* < 0.05), or immune‐related including high density of CD3^−^CD57^+^ NK cells and PD‐L1 combined positive score ≥5 (*p* < 0.05). The best prognostic predictors were a combination of Cyclin A, Cyclin E, p21, and HER3 (*p* < 0.001).

**Conclusion:**

HER2‐positive GC with favorable response to trastuzumab were characterized by cell cycle‐related gene alterations and increased CD3^−^CD57^+^ NK cell infiltration. These findings would be helpful to the fine modulation of therapeutic strategies for patients with HER2‐positive GC.

## Graphical abstract

The molecular and immune microenvironment characteristics of HER2‐positive gastric cancer related to favorable response to first‐line trastuzumab‐based treatment were cell cycle‐related gene alterations including *CCNE1* amplification, increased CD3^‐^CD57^+^ NK cell infiltration, and increased PD‐L1 combined positive score. These findings would be helpful to the fine modulation of therapeutic strategies for patients with HER2‐positive GC.

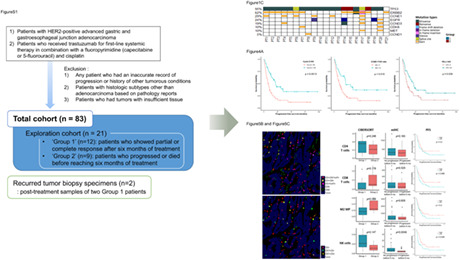

## 1 INTRODUCTION

Gastric cancer (GC) is the third most common cause of cancer‐related mortality worldwide, with the highest incidence in East Asian countries. Human epidermal growth factor receptor 2 (*HER2*), a member of the HER family, is a proto‐oncogene that encodes a receptor tyrosine kinase involved in cell growth and survival.^1^, ^2^ Overexpression of HER2 is a frequent molecular alteration in GC, which is currently an FDA‐approved biomarker to guide first‐line targeted therapy for inoperable locally advanced, recurrent, or metastatic gastric adenocarcinoma.^3^

Trastuzumab, a monoclonal antibody that targets the extracellular domain of HER2, is the first targeted therapy for HER2‐positive GC. Trastuzumab acts primarily by physically inhibiting the dimerization of HER2 with itself or with other HER family receptors. Another proposed mechanism of action is the inhibition of cell proliferation by interfering with cell cycle proteins at the G1/S checkpoint. Through stabilization of p27, trastuzumab inhibits CDK2 and decreases the kinase activity of cyclin E, resulting in cell cycle arrest. Moreover, preclinical studies have suggested that trastuzumab assists the immune system in eliminating tumor cells.^2^, ^4^, ^5^

Trastuzumab has demonstrated proven efficacies against HER2‐positive GC; however, resistance to trastuzumab occurs frequently, limiting its overall benefit in survival outcome.^6^ To understand and overcome this barrier, the resistance mechanisms to HER2‐targeted therapy have come under increasing research attention. HER2 heterogeneity^6^, ^7^ and loss of HER2 expression during trastuzumab treatment^8^ appear to be important factors in HER2‐positive GC. In addition, lower *HER2* amplification levels,^9^ co‐amplification of *HER3*, *CCNE1*, *EGFR* or *MET*,^10^, ^11^, ^12^ and co‐alteration in the downstream RTK/RAS/RAF pathway and PI3K/AKT/mTOR pathway molecules has also been demonstrated to be associated with trastuzumab resistance in GC. However, clinically applicable biomarkers for detecting potential resistant tumors are yet to be discovered.

This study aimed to reveal molecular and immune microenvironment characteristics of HER2‐positive GC according to response to trastuzumab‐based treatment, and to identify biomarkers that could predict trastuzumab resistance by analyzing the genetic alterations and gene expression profiles associated with worse progression‐free survival (PFS) in HER2‐positive GC.

## 2 MATERIALS AND METHODS

### 2.1 Study design

A total of 83 patients with HER2‐positive advanced gastric and gastroesophageal junction adenocarcinomas were retrospectively selected from the archives of Seoul National University Bundang Hospital and Seoul National University Hospital from 2004 to 2018. Patients who received trastuzumab for first‐line systemic therapy in combination with a fluoropyrimidine (capecitabine or 5‐fluorouracil) and cisplatin were included. Clinical data including PFS, defined as the time from the date of trastuzumab treatment to the date of disease progression, death, discontinuation due to adverse effects, or last follow‐up, were collected. Exclusion criteria were as follows: (1) any patient who had an inaccurate record of progression or history of other tumorous conditions, (2) cases with histologic subtypes other than adenocarcinoma based on pathology reports, or (3) cases with insufficient tissue. (Figure S1) The clinicopathological features of the total cohort (*n* = 83) are summarized in Table S1. Most patients were men (81.9%) aged <65 years (57.8%). The histologic subtype of GC was predominantly intestinal (69.9%). The median follow‐up was 22.3 (range 0.7–97.8) months. During the follow‐up period, GC progressed in 58 patients (69.9%) after receiving trastuzumab treatment with a median PFS of 12.4 months.

A subset of the total cohort (hereinafter referred to as exploration cohort, *n* = 21) with sufficient well‐preserved tissue was selected for molecular analysis. Patients of the exploration cohort received trastuzumab treatment only after surgical resection of GC. To demonstrate the differential molecular characteristics based on trastuzumab treatment response, we compared patients who showed partial or complete response after 6 months (‘Group 1’, *n* = 12) with patients who progressed or died before reaching 6 months (‘Group 2’, *n* = 9) of treatment.^13^, ^14^, ^15^, ^16^ Recurred tumor biopsy specimens (post‐treatment samples) of two Group 1 patients were also included for molecular analysis.

### 2.2 Library preparation and analysis of targeted deep sequencing

Targeted deep sequencing was performed on the exploration cohort and post‐treatment samples. All samples that passed quality control were subjected to library preparation using the TruSight Oncology 500 DNA Kit (Illumina) and were sequenced on a NextSeq 550 platform (Illumina), following the manufacturer's instructions.

In brief, the raw sequence reads were converted to FASTQ format using the BaseSpace TSO 500 Assessment App (Illumina) and were aligned to the hg19 genome. The variant call format files were analyzed for single‐nucleotide variants (SNVs) and indels/duplications, and then calculated for tumor mutational burden (TMB). The minimum read depth for reference calls was 100, and the limit of detection for variant allele frequency was 0.05 at that depth. Greater detail on targeted deep sequencing is provided in the Data S1 and sequencing coverage and quality statistics in Table S2A.

### 2.3 Preparation and analysis of RNA sequencing (RNA‐seq)

RNA‐Seq data of the exploration cohort and post‐treatment samples matched to normal tissues were analyzed. In brief, total RNA was extracted using the RNAiso Plus kit (Takara Bio). After purification, tumor RNAs with RNA integrity number ≥6 were subjected to RNA‐seq library preparation using a TruSeq RNA Sample Preparation Kit (Illumina). The synthesized cDNA libraries were sequenced on HiSeq 2000 (Illumina). Copy number variants (CNVs) were called using the CRAFT software and trimmed reads from the Illumina™ FASTQ format were mapped to the human reference genome (GRCh37/hg19) using HISAT2 (v.2.1.0.). Calculated Fragments per kilobase of transcript per million (FPKM) values were converted to transcripts per million. The StringTie results were used to compare the differentially expressed genes (DEGs) between groups. Up‐ and down‐regulated genes with a |log2 fold change| > 2 and *p*‐value <0.01 were selected. Greater detail on RNA‐seq is provided in the Data S1 and sequencing coverage and quality statistics in Table S2B.

### 2.4 Identification of tumor‐infiltrating immune cells from RNA‐seq data

The relative cell fraction and type of immune cells in the tumor microenvironment were estimated using the deconvolution approach. The cell‐type identification by estimating relative subsets of RNA transcripts (CIBERSORT) analytical tool was used to quantify different immune cell types from the RNA‐seq data.^17^ The log‐2 transformed FPKM expression data of the exploration cohort samples were used as the input data source. The LM22 leukocyte gene signature data, including T cell subsets, B cell subsets, monocyte subsets, plasma cells, and NK cells, were used as the input gene signature. For each sample, the calculated immune cell fraction was obtained and expressed as relative quantities.

### 2.5 Gene set enrichment analysis (GSEA)

We performed GSEA; (http://www.broadinstitute.org/gsea/index.jsp) using the KEGG pathways. The gene sets were downloaded from the Molecular Signatures Database (https://www.gseamsigdb.org/gsea/msigdb/genesets.jsp). For each analysis, the number of permutations was set to 1000, and a false discovery rate (FDR) of <0.25 was considered statistically significant.

### 2.6 Tissue microarray (TMA) construction

Next, we constructed TMA from formalin‐fixed paraffin‐embedded (FFPE) samples of the total cohort for FISH and immunohistochemistry (IHC) analyses (SuperBioChips Laboratories). Post‐treatment samples were excluded due to tissue insufficiency. One representative core (2 mm) was selected for each case.

### 2.7 *CCNE1* fluorescence in situ hybridization (FISH)

FISH using the constructed TMA was performed to detect amplification of the *CCNE1* gene using the CCNE1/CEN19p FISH probes (cat. # FG0013, Abnova), according to manufacturer's instructions. Both CCNE1 (orange) and CEP19 (green) signals were evaluated in 40 tumor cells per sample. The average *CCNE1* signal number was calculated, and a CCNE1/CEP19 ratio of ≥2 was considered positive.

### 2.8 IHC of cell cycle‐ and cell proliferation‐related proteins

The BenchMark XT automated slide processing system (Ventana Medical Systems) was used for IHC of cell cycle‐ and cell proliferation‐related proteins on the TMA, following the manufacturer's instructions. The primary antibodies tested are listed in Table S3. One case in Group 2 was excluded due to tissue insufficiency.

Evaluation of the IHC stained slides was based on the H‐score of nuclear expression (cyclin A, cyclin D1, cyclin D3, cyclin E, CDK6, NRG1, and PLK1) or followed established conventional methods. EGFR and HER3 staining were graded according to the HER2 criteria for GC.^3^ p53 was evaluated as null‐type, wild‐type, or overexpression, and p27, p21, and RB as binary criteria (positive or negative). The ki‐67 index was scored as a percentage. Cutoffs for binary assessment were set based on PFS analysis.

PD‐L1 expression was evaluated by combined positive score (CPS) as proposed in a previous study.^18^ CPS cutoffs of 1 and 5 were used for analysis.

### 2.9 Multiplex immunohistochemistry (mIHC) for tumor‐infiltrating immune cells

mIHC analysis was performed using the antibodies listed in Table S3 as described previously.^19^, ^20^ In brief, pre‐processed FFPE TMA slides were first incubated with Harris hematoxylin for nuclear staining, and then subjected to sequential IHC and image acquisition for each primary antibody, as detailed in Data S1. TMA cores were extracted from the acquired images using Aperio ImageScope (Leica Biosystems). CellProfiler ver. 3.1.8 (Broad Institute) was used to perform image alignment and estimate single‐cell staining intensity and cell density.

### 2.10 Statistical analysis

All statistical analyses were performed using R software (version 4.1.0, R Foundation for Statistical Computing). Fischer's exact test and Mann–Whitney test were performed to compare clinicopathological parameters and IHC values. The correlation between the *CCNE1* FISH ratio, RNA data and IHC H‐score was estimated using Spearman rank correlation. Kaplan–Meier curves were constructed for PFS analysis, and statistical significance was assessed using the log‐rank test. All statistical tests were two sided, and a *p*‐value of <0.05 was considered statistically significant.

## 3 RESULTS

### 3.1 Genetic alterations detected by targeted deep sequencing

**FIGURE 1:**
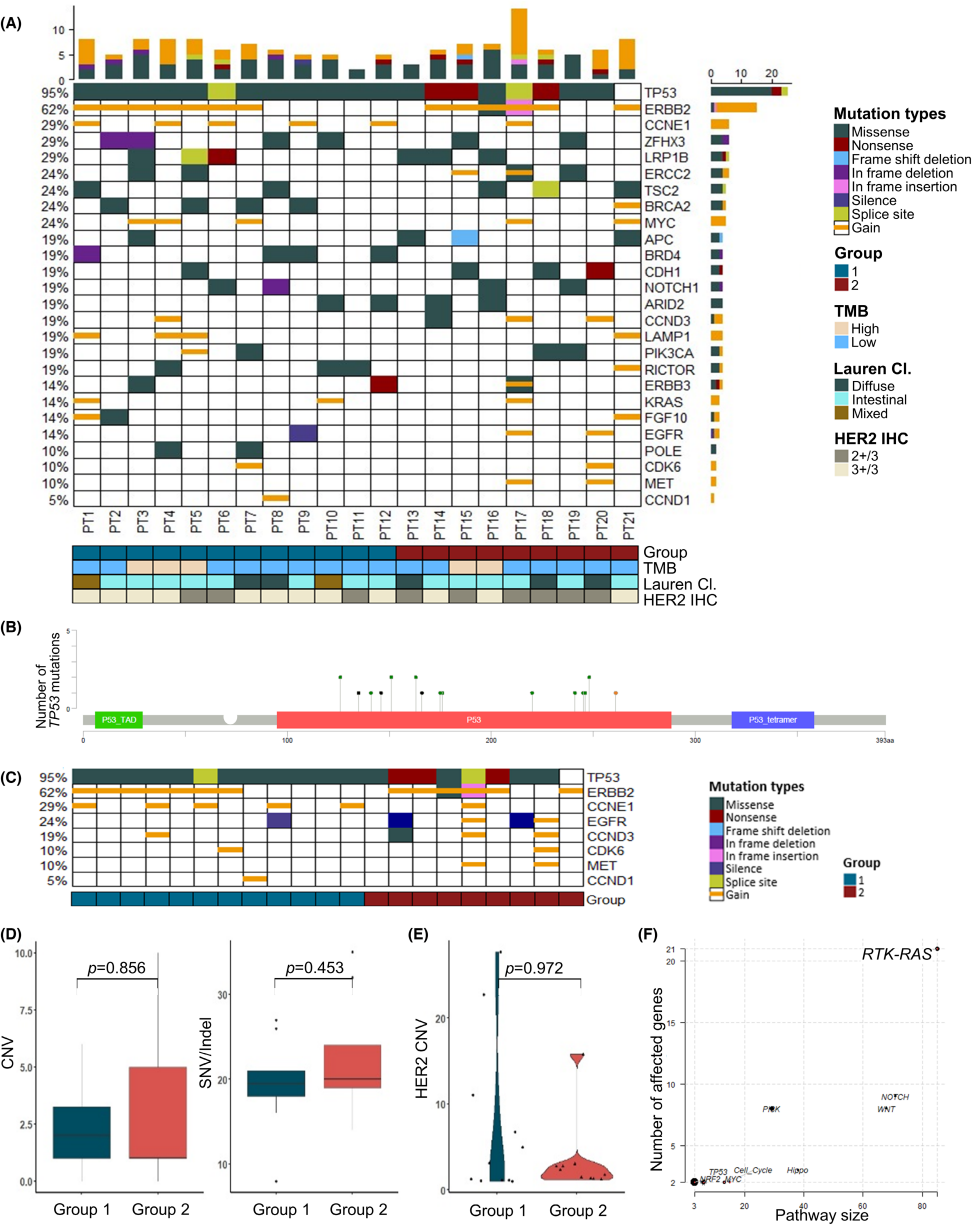
Mutational landscape of HER2‐positive gastric adenocarcinoma by targeted deep sequencing. Oncoplot of targeted deep sequencing in the exploration cohort (A). Lollipop plot of the most frequently mutated gene, *TP53* (B). Altered genes known to be related to trastuzumab resistance and G1/S cell cycle are separately depicted (C). Comparison of CNV and SNV/indel (D), and *HER2* copy number (E) according to progression status at 6 months' period of trastuzumab treatment. Affected oncogenic pathways among known oncogenic signaling pathways in TCGA cohorts (F). *p*‐value by Mann–Whitney test. CNV, copy number variation; IHC, immunohistochemistry; Lauren Cl., Lauren Classification; SNV, single nucleotide variation; TMB, tumor mutation burden.

Targeted deep sequencing revealed that *TP53* (95%) was the most frequently altered gene of the exploration cohort, followed by *ERBB2* (62%), *CCNE1* (29%), *ZFHX3* (29%), and *LRP1B* (29%) (Figure 1A). All tumors in Group 1 (12/12, 100%) and most in Group 2 (8/9, 88.9%) had *TP53* alterations, which were non‐recurrent mutations located in the DNA‐binding domain (Figure 1B). Of these alterations, nonsense mutations were found only in Group 2 (3/9, 33.3%).

Frequent amplification was detected in genes related to the G1/S cell cycle checkpoint (Figure 1C), including *CCNE1* (6/21, 28.6%), *CCND3* (3/21, 14.3%), *CDK6* (2/21, 9.52%) and *CCND1* (1/21, 4.76%). *CCNE1* amplification was observed predominantly in Group 1 (5/12, 41.7% vs. 1/9, 11.1%). Moreover, the overall amplification of the G1/S checkpoint‐related genes was higher in Group 1 than that in Group 2. The total CNV or SNV/indels showed no difference between groups (Figure 1D).

Difference in the amplification of HER family genes were detected between Group 1 and Group 2. *EGFR* (2/21, 9.52%), *MET* (2/21, 9.52%), and *HER3* (1/21, 4.76%), were amplified only in Group 2 (Figure 1C). *HER2* copy number (CN) gain showed little difference between the two groups (mean CN 3.43 vs. 6.96, *p* = 0.972; Figure 1E).

By enrichment of known oncogenic signaling pathways in TCGA cohorts,^21^ the RTK‐RAS signaling pathway was the most affected, followed by the NOTCH and WNT signaling pathways (Figure 1F). Various genes of the RTK‐RAS pathway were altered, including *HER2* (13/21, 61.9%), *HER3* (3/21, 14.3%) and *ALK* (3/21, 14.3%). There was no significant difference between the two groups in the number of genes altered or in the RNA expression of the RTK‐RAS pathway and PI3K/AKT/mTOR pathway (Figure S2).

### 3.2 Association of treatment response with cell proliferation and immune system‐related pathways by transcriptome analysis

**FIGURE 2:**
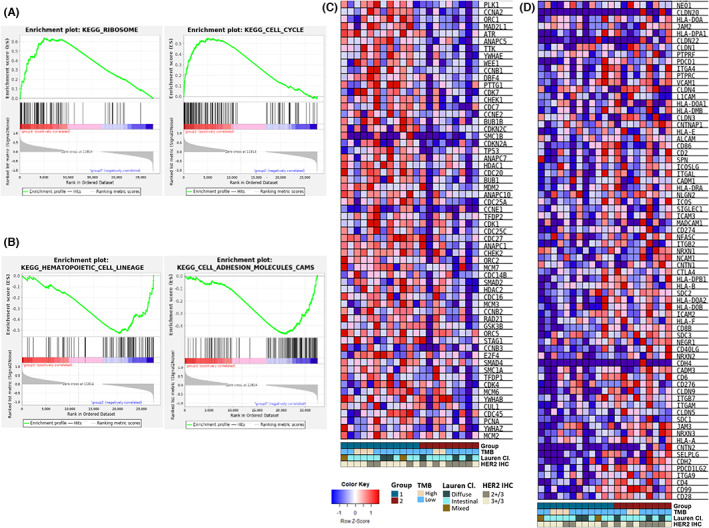
RNA expression and gene sets enrichment analysis (GSEA) of KEGG gene sets. Representative enrichment plots of significant KEGG pathways upregulated in Group 1 compared to Group 2 (A) and in Group 2 compared to Group 1 (B). Leading edge gene expression heatmaps of the ‘Cell cycle’ (FDR *q*‐value 2.43 E‐04, FWER *p*‐value 0.001) (C) and the ‘Cell adhesion molecules CAMS’ (FDR *q*‐value 0.01, FWER *p*‐value 0.037). (D). FDR, false discovery rate; FWER, familywise‐error rate; Lauren Cl., Lauren classification; TMB, tumor mutation burden.

A total of 113 DEGs were found in the two groups. The significant KEGG pathways identified by GSEA are listed in Table S4 and the representative KEGG pathways of each group is depicted in Figure 2. Compared with Group 2, the significantly upregulated DEGs in Group 1 were mostly enriched in KEGG pathways related to cell proliferation and growth, such as the KEGG ‘Cell cycle’ pathway (Figure 2A,C) In contrast, KEGG pathways enriched in Group 2 were mainly related to the immune system. Leading edge genes of significant pathways included genes related to immune suppression, such as *CD274*, *CD276*, and *CTLA4* (Figure 2B,D).

### 3.3 IHC and *CCNE1* FISH validates genomic and transcriptomic sequencing results

**FIGURE 3:**
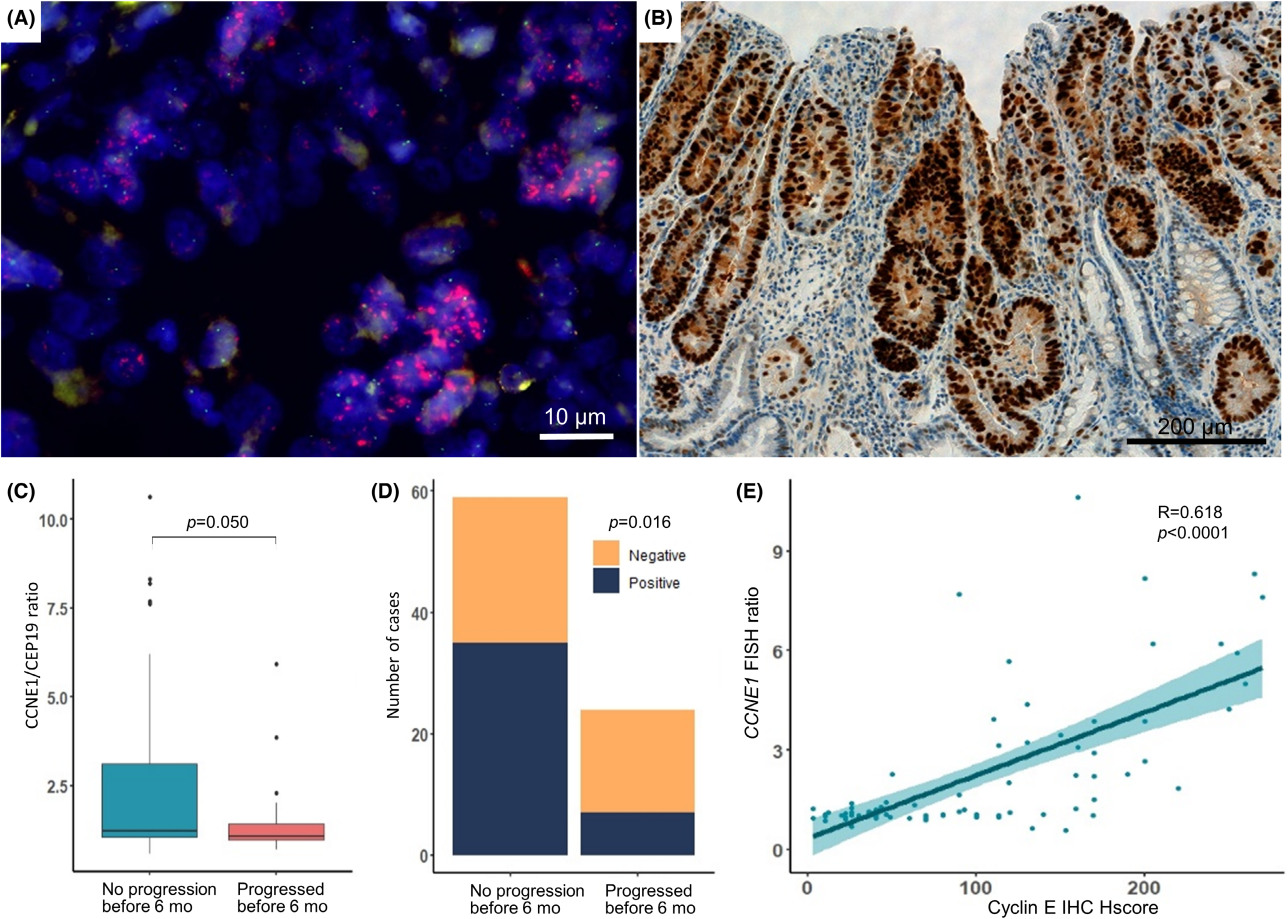
Fluorescence in situ hybridization (FISH) and immunohistochemistry (IHC) of cases with *CCNE1* copy number gain. All cases with *CCNE1* copy number gain were tested for and confirmed positive by both *CCNE1* FISH (A, ×1000) and IHC (B, ×200). Significantly higher *CCNE1* gene amplification by FISH CCNE1/CEP19 ratio (C) and Cyclin E protein expression (D) were observed in patients who did not progress before 6 months than those who did (*p* = 0.050 and 0.016, respectively). The *CCNE1* FISH ratio and IHC showed good positive correlation (*r* = 0.68, *p* < 0.001). (E) *p*‐value by Mann–Whitney test and Fisher‐exact test. Correlation coefficient rho (*R*) by Spearman rank correlation. Mo, months.

Group 1 showed frequent amplification of *CCNE1* by targeted deep sequencing and enrichment in KEGG pathways related cell proliferation and growth. Therefore, to confirm the preferential presence of *CCNE1* amplification in Group 1, we performed FISH and IHC for *CCNE1*. In the exploration cohort, all six cases with *CCNE1* CN gain (Figure 1C) confirmed to be *CCNE1*‐amplified by FISH with high *CCNE1* signal numbers (median of average signals 8.67 [4.68–15.58]) (Figure 3A). FISH detected an additional case in Group 2 to have *CCNE1* amplification (CCNE1/CEP19 ratio 2.28), formerly not found by targeted deep sequencing. Positive protein expression of cyclin E (Figure 3B) was observed in all the FISH‐confirmed *CCNE1‐*amplified cases. RNA expression data also correlated positively with *CCNE1* FISH ratio (*r* = 0.78, *p* < 0.001), FISH average signal number (*r* = 0.79, *p* < 0.001) and cyclin E protein H‐score (*r* = 0.72, *p* < 0.001).

In the total cohort, FISH analysis revealed 31.3% (26/83) *CCNE1* positivity and cyclin E expression was observed in 51.2% (42/82). When comparing patients who progressed or died before 6 months of trastuzumab treatment and those who did not in the total cohort, the former showed a lower tendency of CCNE1/CEP19 ratio (median 1.0 [0.9–1.4] vs. median 1.2 [1.0–3.1], *p* = 0.050; Figure 3C) and weaker expression of Cyclin E protein (7/23, 30.4% vs. 35/59, 59.3%, *p* = 0.016) (Figure 3D). A positive correlation was found between *CCNE1* FISH ratio and cyclin E IHC (*r* = 0.618, *p* < 0.001; Figure 3E).

In addition, the leading edge genes in KEGG ‘Cell cycle’ pathway were further investigated by IHC (Figure S3) because proteins regulating the cell cycle drive cell proliferation and trastuzumab interferes with the G1/S checkpoint. Cases with CN gain of cell cycle‐related genes by targeted deep sequencing were confirmed to overexpress proteins of the respective gene product (*CCND3 p* = 0.014 and *CDK6 p* = 0.004), except for *CCND1* (*p* = 0.142). Notably, every case with *TP53* nonsense mutation showed p53 null‐type expression. In the total cohort, the expression profiles of cell cycle‐related proteins according to treatment response revealed significantly lower expression of cyclin A (1/23, 4.35% vs. 15/59, 25.4%, *p* = 0.032), PLK1 (16/23, 69.6% vs. 48/59, 81.4%, *p* < 0.001) and p21 (2/23, 30.4% vs. 19/59, 59.3%, *p* = 0.046) in the patients who progressed or died before 6 months (Figure S4).

### 3.4 Expression profile of a combination of cell cycle‐related proteins predict treatment outcomes

**FIGURE 4:**
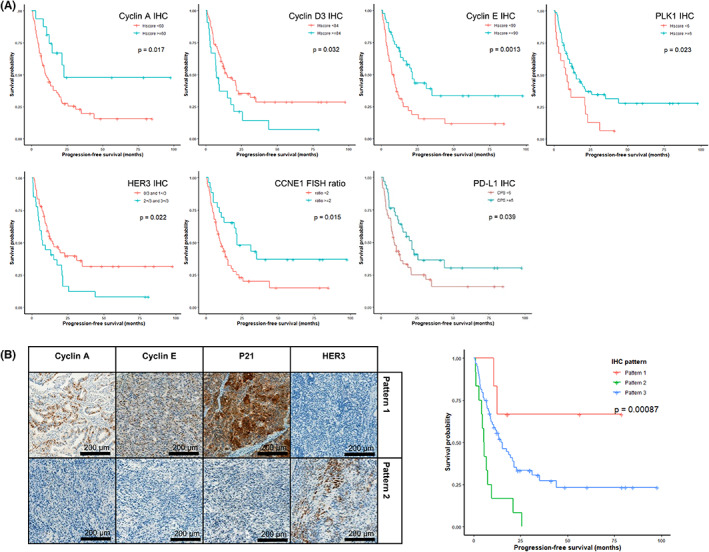
Progression‐free survival analysis of HER2 positive gastric adenocarcinoma. Kaplan–Meier curves with significant difference (*p* < 0.05) are shown (A). Expression patterns of selected cell cycle proteins can predict progression‐free survival (*p* < 0.001) (B). Cell cycle proteins that significantly predicted PFS or showed significant difference between Group 1 and Group 2 were selected.

Significant differences in PFS were observed with IHC of cyclin A (*p* = 0.017), cyclin D3 (*p* = 0.032), cyclin E (*p* = 0.001), PLK1 (*p* = 0.023), and HER3 (*p* = 0.022), along with *CCNE1* FISH ratio (*p* = 0.015; Figure 4A). The IHC marker combination that could best predict trastuzumab treatment response was cyclin A, cyclin E, and p21, and HER3 (*p* < 0.001; Figure 4B). Positive expression of cyclin A, cyclin E, and p21, in addition to negative expression of HER3 (Pattern 1), showed the best PFS (median PFS 18.0 months). In contrast, the direct opposite expression profile (Pattern 2) represented the worst prognosis (median PFS 5.45 months). All other combinations of expression were included in Pattern 3 (median PFS 15.3 months).

Furthermore, positive PD‐L1 expression, represented by CPS ≥1 and CPS ≥5, was identified in 61.0% (50/82) and 41.5% (34/82) of all cases, respectively. None of the tumors showed more than 1% of tumor cell PD‐L1 expression. PD‐L1 expression stronger than CPS of 5 was significantly associated with favorable PFS (*p* = 0.039) (Figure 4A).

### 3.5 Characterization of the immune microenvironment by CIBERSORT analysis and mIHC

**FIGURE 5:**
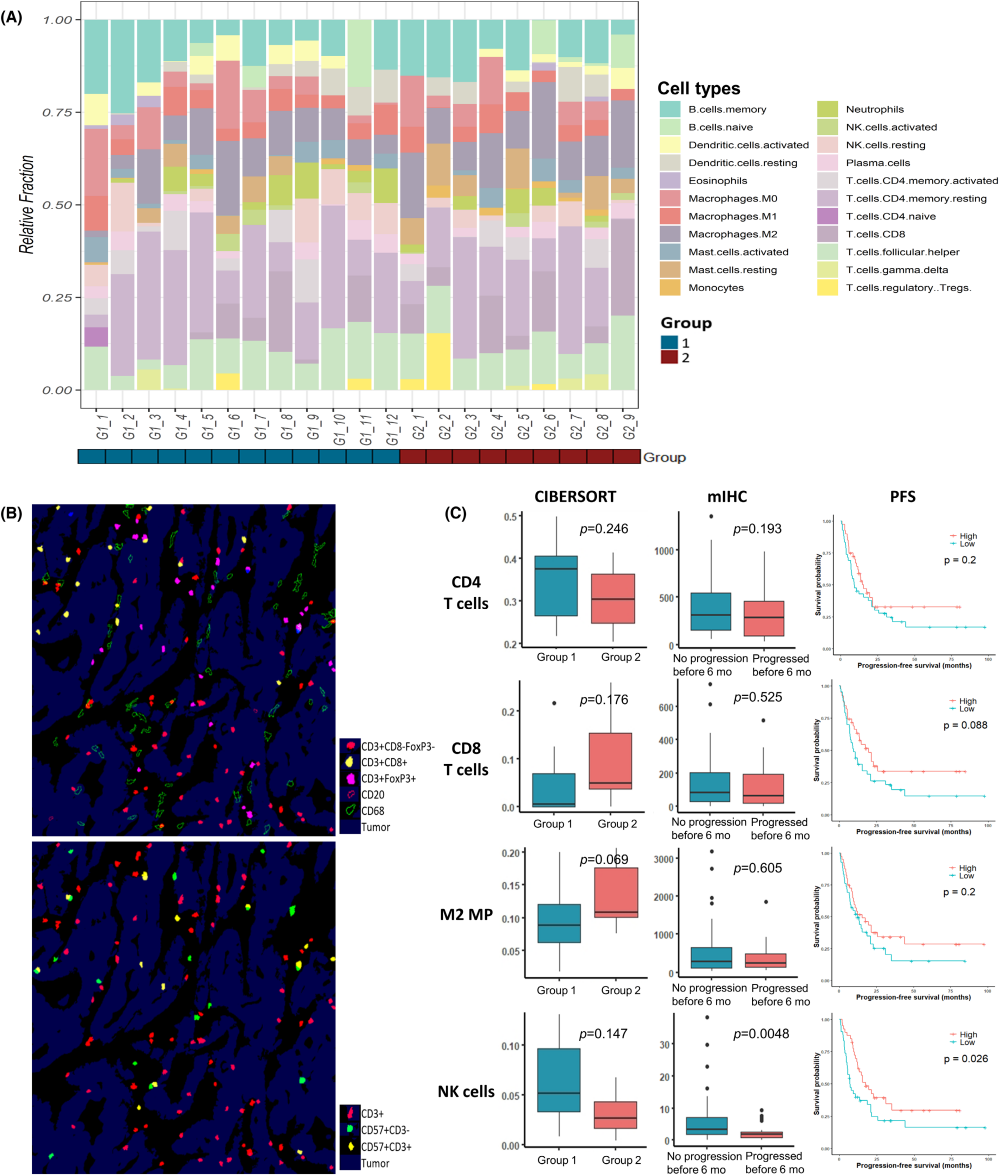
Tumor infiltrating immune cells analyzed by CIBERSORT in the exploration cohort (A) and multiplex immunohistochemistry (mIHC) in the total cohort (B). Comparison of CIBERSORT and mIHC evaluations of CD4 T cells, CD8 T cells, M2 macrophages and NK cells (C) reveal that patients who did not progress before 6 months have significantly more NK cell infiltration than those who did (*p* = 0.0048 by mIHC). CD3^−^CD57^+^ NK cells contributed to better progression‐free survival in the total cohort (*p* = 0.026). *p*‐value by Mann–Whitney test. M2 MP, M2 macrophage.

The GSEA results revealed upregulation of immune system‐related pathways in Group 2 than in Group 1, which was further validated by analyzing the RNA‐seq data using the CIBERSORT tool (Figure 5A). Across the exploration cohort, the immune cells mainly comprised of CD4 memory resting T cells, follicular helper T cells, memory B cells, and M2 macrophages in decreasing order. Furthermore, Group 1 tended to have more NK cells and CD4 T cells, while Group 2 was infiltrated more by M2 macrophages and CD8 T cells, although without significance (*p* = 0.147, 0.246, 0.069, and 0.176, respectively) (Figure 5C).

Tumor‐infiltrating immune cells were assessed by mIHC using IHC markers for CD4 T cells, CD8 T cells, regulatory T cells, NK cells, M2 macrophages, and B cells (Figure 5B). mIHC revealed that CD3^−^CD57^+^ NK cells infiltrated tumors of the patients with prolonged PFS significantly more than those with shorter PFS (*p* = 0.0048) (Figure 5C). By Kaplan–Meier analysis, the CD3^−^CD57^+^ NK cells also contributed to a better PFS (*p* = 0.026). In addition, stronger PD‐L1 expression (CPS ≥5) was associated with higher CD8 T cell, CD4 T cell, and M2 macrophage infiltration (*p* = <0.001, 0.001, <0.001, respectively). However, significant difference in densities of CD4 T cells, CD8 T cells, and M2 macrophages between the two groups were not found by mIHC.

### 3.6 Genomic and transcriptomic landscape changes during trastuzumab treatment

**FIGURE 6:**
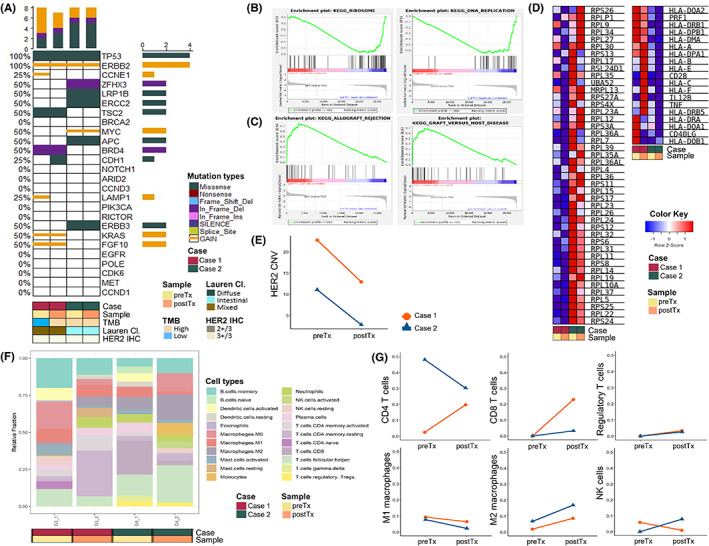
Genomic and transcriptomic landscape change of gastric cancer during trastuzumab treatment. Targeted deep sequencing shows little genetic difference during treatment (A). GSEA result of pre‐treatment samples compared to post‐treatment samples (B) and that of post‐treatment samples compared to pre‐treatment samples (C). RNA expression of leading edge genes of the KEGG ‘Ribosome’ and ‘Allograft rejection’ pathway (D). HER2 copy number decreases after trastuzumab treatment (E). CIBERSORT reveals a tendency of increased CD8 T cell and M2 macrophage fraction during treatment (F, G). CNV, copy number variation; Lauren Cl., Lauren classification; postTx, post‐treatment; preTx, pre‐treatment; TMB, tumor mutational burden.

In Group 1, two patients who progressed after 6‐months—one (Case 1) at 21 months and the other (Case2) at 11 months of treatment—were subjected to an additional biopsy for the recurrent metastatic tumor in the peritoneal seeding and liver, respectively. The recurred tumor biopsy specimens were tested alongside the exploration cohort for genomic and transcriptomic analysis (Figure 6). Targeted deep sequencing revealed loss of *CCNE1* and *LAMP1* amplifications in one case (Figure 6A), and decreased HER2 CN in both cases (Figure 6E).

RNA‐seq revealed 25 DEGs, most upregulated in post‐treatment samples compared with pre‐treatment samples. GSEA found upregulated DEGs in pre‐treatment samples enriched in KEGG pathways related to cell proliferation and growth. Those in post‐treatment samples were enriched in immune system‐associated pathways (Table S4; Figure 6B–D).

Furthermore, investigation of the changes in immune cell composition before and after treatment revealed increased CD8 T cells and M2 macrophages and a decreased fraction of M1 macrophages in post‐treatment samples than those in pre‐treatment samples (Figure 6F,G).

## 4 DISCUSSION

This study compared the genomic and transcriptomic profiles of patients with HER2‐positive GC in a PFS period of 6 months after trastuzumab therapy. The study demonstrated that HER2‐positive GC is characterized by *TP53* mutation, frequent CN gain, and upregulation of cell proliferation‐related genes at the DNA, RNA, and protein levels. Further, it provided evidence that lower levels of *HER2* CN, cell cycle‐related gene expression, and NK cell count are the crucial factors that diminish the trastuzumab effect. In contrast, strong cell cycle protein expression, particularly cyclin E, and higher PD‐L1 expression and NK cell infiltration, were associated with better PFS.

HER2‐positive GC responsive to trastuzumab showed frequent amplification and overexpression of cell cycle‐related genes, including *CCND1, CCNE1, CCND3*, and *CDK6*. In particular, cyclin E promotes cell proliferation by acting on the G1/S checkpoint, where trastuzumab suppresses the transition of the cell cycle. *CCNE1* is frequently co‐amplified in 11%–40% of HER2‐positive GC.^6^, ^22^, ^23^ In this study, patients with GC showing *CCNE1* amplification and strong cyclin E expression benefited more from trastuzumab treatment. These results are concordant with a few previous studies. For example, Takano et al.^24^ investigated the overexpression of cell cycle proteins in GC and found that cyclin E overexpression correlates with tumor differentiation and good prognosis.

In contrast, many studies have argued opposing results. Lee et al.^10^ explored trastuzumab response in HER2‐positive GC following a similar approach to dichotomize patients, albeit with a PFS period of 8 months. The study demonstrated that *CCNE1* amplification was a significant predictor of shorter PFS, while EGFR overexpression had no prognostic significance. In addition, the association of *CCNE1* amplification in GC with liver metastasis,^25^ treatment resistance^23^ and short survival,^6^, ^26^, ^27^ have also been reported. Considering previous studies, our study suggests that further research is essential to confirm the effect of *CCNE1* amplification on the treatment outcomes of HER2‐positive GC.

The immune system also participates in the therapeutic effects of trastuzumab. This study demonstrated that CD57^+^CD3^−^ NK cells contributes to favorable PFS. The potential of NK cell utilization in cancer immunotherapy is recently under active investigation.^28^, ^29^ CD57^+^CD3^−^ NK cells are mature NK cells that possess cytotoxic ability.^2^, ^30^ NK cells recognize Fc receptors of antibodies attached to target cells and lyse the antibody‐coated cells, a process known as antibody‐dependent cellular cytotoxicity (ADCC). Research on breast cancer has discovered that NK cells recognize anti‐HER2 antibodies, and that ADCC is a major mechanism of action for trastuzumab.^4^, ^29^ Trastuzumab‐mediated ADCC also contributes to tumor cell death in HER2‐positive GC cells^5^, ^31^, ^32^ but is impaired in patients with advanced disease.^33^ Our study also supports that diminished NK cell‐mediated ADCC is an influencing factor for the lack of trastuzumab treatment response.

Furthermore, the main regulation mechanism of tumor immune microenvironment may contribute to the difference in PFS. HER2‐positive GCs with better PFS were associated with stronger PD‐L1 expression (CPS ≥5) in addition to higher CD3‐CD57+ NK cell infiltration. The PD‐L1 protein in GCs of this study were mostly expressed in tumor‐infiltrating immune cells and strong PD‐L1 expression was associated with higher infiltration of M2 macrophage, CD8 T cells, and CD4 T cells. Previous in vitro studies demonstrated that trastuzumab‐sensitive tumors recruit immune cells by secretion of chemokines and exhibit higher levels of PD‐1 ligands.^34^ This implies that HER2‐positive GC that are immune hot tumors under immunosuppression by the PD‐1/PD‐L1 axis shows improved outcome by trastuzumab treatment. Therefore, early introduction to combined treatment of trastuzumab and immunotherapy, such as pembrolizumab, may show benefit in the objective response rate of HER2‐amplified GC, as was recently demonstrated in the KEYNOTE‐811 trial.^35^

On the other hand, HER2‐positive GCs with poor PFS appear to have additional immune evasion mechanisms besides the PD‐1/PD‐L1 axis. GSEA analysis revealed upregulated leading edge genes such as *CD276* and *CTLA4* in immune‐related KEGG pathways. *CD276* (B7‐H3) inhibits T cell proliferation and NK cell‐mediated cytotoxicity,^36^ while *CTLA4* suppresses the function of T cells. Both act synergistically with the PD‐1/PD‐L1 axis.^37^ Immunotherapy using dual checkpoint inhibitors currently being explored^38^ could be beneficial for these patients.

Previously established resistance mechanisms to trastuzumab were also present in our study. The infamous heterogeneity of HER2 in GC,^6^, ^7^ resulted in the HER2 concordance rate of 62% between IHC/SISH and targeted deep sequencing results. Discordant cases were markedly heterogeneous, showing IHC intensity of 2+ or 3+ in 5%–20% of the tumor areas (Figure S5). Moreover, the level of *HER2* amplification and decrease of *HER2* CN during treatment demonstrated in this study has been reported to predict treatment response and prognosis in GC.^1^, ^6^, ^8^, ^9^ Lastly, amplification of *EGFR*, *HER3* and *MET*, and strong HER3 expression was associated with poor PFS. These results support previous observations^4^, ^6^, ^12^, ^39^ that the HER family overexpression can contribute to trastuzumab resistance by alternative signaling.

In addition to the known resistance factors, our study also demonstrated that, although *TP53* mutation is the most frequent mutation in HER2‐positive GC, *TP53* nonsense mutations occurred mainly in treatment‐resistant cases. However, p53 null‐type expression did not show significant difference in PFS. Therefore, nonsense mutations should be confirmed in the null‐type expressed cases to unveil the impact of p53 loss on trastuzumab treatment effect.

A shift in RNA expression and immune cell composition was noted in GC before and after trastuzumab treatment. GSEA revealed common KEGG pathways between pre‐treatment samples and Group 1 and between post‐treatment samples and Group 2. In post‐treatment samples, the change in M2 macrophages and CD8 T cells signified a similar trend of immune cell composition as that in Group 2. These alterations suggest a shift toward resistant characteristics, rendering the tumor withstand treatment.

Resistance mechanisms can overlap between therapeutic agents while any drug can induce multiple factors of resistance. Different predictive markers have been suggested for current therapeutic agents of GC^40^ implying that specific genetic and immune microenvironment characteristics of GC may occur according to the drug treated. Although we have not been able to ascertain with experiments ourselves, according to previous studies^40^, ^41^, ^42^ cell cycle‐related or HER‐family gene amplifications seem to be rather specific changes of HER2‐targeting agents in comparison to antiangiogenic agents or immunotherapeutic drugs. On the other hand, the immune composition characteristics of GC still remains to be elucidated,^43^, ^44^ and further research is warranted for validation of the predictive value of NK cells on HER2‐positive GC treated with trastuzumab.

In order to verify and reinforce our findings on RNA‐seq and immune cell population, carefully constructed research using cell‐line or animal assays would be optimal. Although it is a limitation of our study, previous preclinical studies have reported the antitumor activity of NK cells against GC,^31^ immune cell population change in response to chemotherapy,^43^ and HER2 receptor or signaling pathway changes related to HER‐targeted therapies.^41^ However, RNA‐seq and GSEA conducted on gastric cell lines have reported various results^43^, ^45^; thus, further in vitro and in vivo experiments would provide more convincing results. In addition, the exploration cohort of this study included only surgical specimens of treatment naïve HER2‐positive GC. The patients in the exploration cohort received trastuzumab therapy after surgery. Therefore, the results of targeted sequencing and RNA‐seq may indicate intrinsic resistance rather than acquired resistance, while those of the total cohort imply both resistance mechanisms.

This study is unique in that it focuses on characteristics of HER2‐positive GC according to the time to progression after trastuzumab treatment. Furthermore, this study analyzed the whole transcriptome and the immune microenvironment on top of the genetic alterations in the same FFPE specimens, leading to a comprehensive analysis of HER2‐positive GC. Moreover, the results of targeted sequencing and RNA‐seq were further validated by IHC.

In conclusion, this study demonstrates the genetic and immune microenvironment characteristics according to trastuzumab‐based treatment and suggests potential biomarkers to predict PFS of patients with HER2‐positive GC. These findings may help to provide a basis for the fine modulation of personalized therapeutic strategies for HER2‐positive GC. The assessment of cell cycle‐related gene alterations and tumor‐infiltrating immune cells early on treatment can serve as a guide to treatment decisions in utilizing the combination of targeted and immune therapies for maximum treatment response.

## AUTHOR CONTRIBUTIONS

**Hyun Jung Kwon:** Conceptualization (equal); formal analysis (lead); investigation (lead); visualization (lead); writing – original draft (lead); writing – review and editing (equal). **Yujun Park:** Formal analysis (equal); investigation (equal); visualization (equal); writing – review and editing (supporting). **Soo Kyung Nam:** Formal analysis (equal); investigation (equal); visualization (equal); writing – review and editing (equal). **Enoch Kang:** Formal analysis (equal); investigation (equal); visualization (equal); writing – review and editing (supporting). **Ka‐Kyung Kim:** Formal analysis (equal); resources (equal); visualization (equal); writing – review and editing (supporting). **Inhae Jeong:** Formal analysis (equal); visualization (equal); writing – review and editing (supporting). **Yoonjin Kwak:** Formal analysis (supporting); investigation (equal); resources (equal); writing – review and editing (equal). **Jeesun Yoon:** Resources (equal); writing – review and editing (equal). **Tae Yong Kim:** Resources (equal); writing – review and editing (equal). **Keun‐Wook Lee:** Resources (equal); writing – review and editing (equal). **Do‐Youn Oh:** Resources (equal); writing – review and editing (equal). **Seock‐Ah Im:** Resources (equal); writing – review and editing (equal). **Seong‐Ho Kong:** Resources (equal); writing – review and editing (equal). **Do Joong Park:** Resources (equal); writing – review and editing (equal). **Hyuk‐Joon Lee:** Resources (equal); writing – review and editing (equal). **Hyung‐Ho Kim:** Resources (equal); writing – review and editing (equal). **Han‐Kwang Yang:** Resources (equal); writing – review and editing (equal). **Hye‐Seung Lee:** Conceptualization (lead); formal analysis (equal); funding acquisition (lead); investigation (equal); project administration (lead); resources (lead); supervision (lead); writing – review and editing (lead).

## FUNDING INFORMATION

This work was supported by the National Research Foundation of Korea (NRF) grant funded by the Korea government (Ministry of Science and ICT) (No. 2019R1A2C1086180).

## CONFLICT OF INTEREST STATEMENT

The authors declare no competing interests.

## ETHICS APPROVAL AND CONSENT TO PARTICIPATE

This work was approved by the Institutional Review Board from Seoul National University Hospital (J‐1907‐173‐1050). The consent process was waived by the IRB under the condition of anonymization and no additional participant intervention.

## Supporting information


Data S1

Table S1

Table S2

Table S3

Table S4

Figure S1

Figure S2

Figure S3

Figure S4

Figure S5

## Data Availability

The datasets obtained and/or analyzed during the current study are available from the corresponding author upon reasonable request.
