# Supplementary material for: Genetic and immune microenvironment characterization of HER2‐positive gastric cancer: Their association with response to trastuzumab‐based treatment
Source: Cancer Med. 2023 Mar 14;12(9):10371–84. doi: 10.1002/cam4.5769 (PMC10225221; doi:10.1002/cam4.5769)
Supplement: Supplementary file 1 — Data S1 [file CAM4-12-10371-s001.pdf]

Supplementary Methods

Library preparation of next-generation sequencing (NGS)

Targeted genome sequencing was performed on the exploration cohort and post-treatment samples. All samples that passed quality control were subjected to library preparation using the hybrid capture-based TSO 500 library preparation kit TruSight Oncology 500 DNA Kit (Illumina, San Diego, CA, USA) following the manufacturer's instructions. Briefly, the DNA was fragmented using an ultrasonicator (Covaris, Woburn, MA, USA) with a target peak of ~130 bp. After end repair, A-tailing, and adapter ligation, the adapter-ligated fragments were amplified using index PCR (UP-index)-specific primers. Furthermore, the libraries were enriched using a hybrid capture-based method using specific probes followed by PCR-based enrichment, cleanup, and quantification of double-stranded DNA using high-sensitivity Qubit (#Q32854 Invitrogen, USA). The libraries were subjected to bead-based normalization and were sequenced using V2 sequencing reagent kits on a NextSeq 550 platform (Illumina, San Diego, CA), following the manufacturer's recommendations.

Analysis of targeted genomic sequencing data

The raw sequence reads were converted to FASTQ format using the BaseSpace TSO 500 Assessment App (Illumina). The DNA sequences in FASTQ files were aligned to the hg19 genome using the Burrows-Wheeler Aligner with the SAMtools. Pre-processing by read collapsing using a unique molecular identifier (UMI), and Stitching Pisces software was used to detect low frequencies somatic variants in DNA samples. The variant call format (VCF) files were analyzed using the Basespace variant

interpreter for single-nucleotide variants (SNVs) and indels/duplications. The minimum read depth for reference calls was 100, and the limit of detection for variant allele frequency (VAF) was 0.05 at that depth. Post-processing on the small variant calling VCFs was performed using Pepe for background polishing and quality score adjustment. In-house filtering, functional annotation, and reporting were performed using several databases, such as ClinVar, SnpEff, and ExAC. Variants with a VAF <2% or ≥ 97%, AF ≥ 1% in ExAC and Macrogen, were removed for germline filtering, and benign/likely benign variants in the ClinVar database were filtered for clinical significance analysis. Furthermore, non-functional regions such as synonymous, intron, 3' UTR, 5' UTR, upstream, downstream, and intergenic regions were excluded. Tumor mutational burden (TMB) detection was set by counting the eligible SNVs and indels per Mb in the coding regions and high-confidence regions with ≥ 50× coverage. Microsatellite instability status (MSI) using 130 MSI marker sites to calculate a quantitative score was recorded using the Basespace TSO 500 Assessment App. Copy number variants (CNVs) were called by performing amplification, reference, and deletion calling for target CNV genes within the assay using the CRAFT software. The CRAFT software component counts the coverage of each target interval on the panel, performs normalization, calculates fold change values for each gene, and determines the CNV status for each CNV target gene. During the normalization steps, coverage biases were corrected using potential variables such as sequencing depth, target size, PCR duplicates, probe efficiency, GC bias, and DNA type. A collection of normal FFPE and genomic DNA samples was used to correct some of these variables. The gene-specific threshold for amplification and deletion for each target CNV gene on the panel was estimated.

Preparation and analysis of RNA sequencing (RNA-seq)

Total RNA from the exploration cohort and post-treatment samples was extracted using the RNAiso Plus kit (Takara Bio, Shiga, Japan). Extracted RNA was purified using the RNeasy Mini Kit with DNase I (Qiagen) treatment. RNA integrity was assessed using a Bioanalyzer (Agilent, USA), and tumor RNAs with RNA integrity number (RIN) ≥ 6 were subjected to RNA-seq. RNA-seq libraries were generated using a TruSeq RNA Sample Preparation Kit (Illumina, USA). Briefly, mRNA was enriched using poly T oligo-attached magnetic beads, followed by mRNA fragmentation by acoustic shearing. First-strand cDNA was synthesized using reverse transcriptase and random hexamers. Second-strand cDNA was synthesized using DNA polymerase I and RNase H. Subsequently, cDNA was subjected to adapter ligation and then enriched with PCR to prepare a cDNA library. cDNA libraries were sequenced on a HiSeq 2000 (Illumina) to obtain approximately 100 million paired-end reads (2 × 101 bp). Copy number variants (CNVs) were called by performing amplification, reference, and deletion calling for target CNV genes within the assay using the CRAFT software.

RNA-Seq data of 21 patients with HER2-positive gastric cancer matched to normal tissues were analyzed. Briefly, after sequencing, raw data were obtained in the FASTQ format. FastQC was used to validate data quality. Trimming of the adaptor content and over-represented sequences was performed using Trimmomatic v.1. Trimmed reads from the Illumina™ FASTQ format were mapped to the human reference genome (GRCh37/hg19) using HISAT2 (v.2.1.0.) with Ensembl gene annotation and fr-firststrand library type. Fragments per kilobase of transcript per million (FPKM) values were calculated using StringTie (v.1.3.4d) that used the aligned bam files and were

converted to transcripts per million (TPM). After data preprocessing, the StringTie results were used to compare the differentially expressed genes (DEGs) between groups. Up-and down-regulated genes with a |log2 fold change(FC) | > 2 and p-value < 0.01, were selected. A density plot showing the distribution of RNA-seq read counts (FPKM) of Group 1 vs. Group 2 was created using R.

Multiplex IHC (mIHC) for tumor-infiltrating immune cells

mIHC analysis was performed using the antibodies listed in Table 2 as described previously (19, 20). In brief, preprocessed FFPE TMA slides were first incubated with Harris hematoxylin (Merck, Darmstadt, Germany) for nuclear staining and then subjected to sequential IHC and image acquisition for each primary antibody as follows: After antigen retrieval and blocking, TMA slides were incubated with the specific antibody of choice, and then the Envision FLEX + mouse linker/rabbit linker (Dako, Carpinteria, CA, USA) was treated with a secondary reagent. ImmPact NovaRED (Vector Laboratories, Burlingame, CA, USA) was used for chromogenic reaction and visualization. After nuclear conterstaining with Mayer’s hematoxylin (Dako, Carpinteria, CA, USA), images were acquired by whole slide scanning using an Aperio AT2 scanner (Leica Biosystems, Newcastle upon Tyne, UK). The slides were then treated with a stripping buffer (20% sodium dodecyl sulfate, 0.5 M Tris-HCl pH 6.8, β-mercaptoethanol, and distilled water) and microwaved to remove the antibodies.

Each TMA core was extracted from the acquired images using Aperio ImageScope (Leica Biosystems). CellProfiler ver. 3.1.8 (Broad Institute, Cambridge, MA, USA) was used to align the stained images for each TMA core and estimate single-cell staining intensity and cell density. The staining intensity was interpreted as positive or negative

by appropriate cutoffs (19,20). Then, the density of stained cells was calculated as the number of positive cells per mm2.
