# Supplementary material for: Genetic and immune microenvironment characterization of HER2‐positive gastric cancer: Their association with response to trastuzumab‐based treatment
Source: Cancer Med. 2023 Mar 14;12(9):10371–84. doi: 10.1002/cam4.5769 (PMC10225221; doi:10.1002/cam4.5769)
Supplement: Supplementary file 2 — Table S1 [file CAM4-12-10371-s004.pdf]

**Supplementary Table 1.**

| Features | Exploration cohort | | | | | Total | |
|---|---|---|---|---|---|---|---|
| | Group 1 | | Group 2 | | | | |
| | N | % | N | % | *P*-value | N | % |
| **Total** | 12 | 100.0 | 9 | 100.0 | | 83 | 100.0 |
| **Sex** | | | | | | | |
| Female | 1 | 8.3 | 1 | 11.1 | 1.000[a] | 15 | 18.1 |
| Male | 11 | 91.7 | 8 | 88.9 | | 68 | 81.9 |
| **Age** | | | | | | | |
| <65 | 7 | 58.3 | 6 | 66.7 | 1.000[a] | 48 | 57.8 |
| ≥65 | 5 | 41.7 | 3 | 33.3 | | 35 | 42.2 |
| **Lauren classification** | | | | | | | |
| Intestinal type | 8 | 66.6 | 6 | 66.7 | 0.425[a] | 58 | 69.9 |
| Diffuse type | 2 | 16.7 | 3 | 33.3 | | 19 | 22.9 |
| Mixed type | 2 | 16.7 | 0 | 0.0 | | 6 | 7.2 |
| **Progression after starting HER2 treatment** | | | | | | | |
| Before 6 months | 0 | 0.0 | 9 | 100.0 | <0.001[a] | 24 | 28.9 |
| After 6 months | 2 | 16.7 | 0 | 0.0 | | 34 | 41.0 |
| Progression free at last follow-up | 10 | 83.3 | 0 | 0.0 | | 25 | 30.1 |
| **Progression-free survival** (months) | | | | | | | |
| median [range] | 22.5 [11.7-68.1] | | 4.3 [1.7-5.6] | | <0.001[b] | 12.4 [5.3-23.3] | |

[a]Fisher's exact test; [b]Mann-Whitney test
