# Supplementary material for: Genetic and immune microenvironment characterization of HER2‐positive gastric cancer: Their association with response to trastuzumab‐based treatment
Source: Cancer Med. 2023 Mar 14;12(9):10371–84. doi: 10.1002/cam4.5769 (PMC10225221; doi:10.1002/cam4.5769)
Supplement: Supplementary file 3 — Table S2 [file CAM4-12-10371-s006.pdf]

Supplementary Table 2.

(a)

Sample ID (GRCh37)	Total number of sequenced reads	Total number of uniquely mapped non-duplicate reads	Total number of covered bases	Median coverage (and range) per base	Percentage of targeted bases with coverage ≥10 (targeted panel: Illumina TruSight Oncology 500)
S09-0024194	69,872,856	45,681,504	3,830,473,059	270 (1-9910)	99.7
S15-0005814	76,888,782	50,030,328	4,229,313,877	380 (1-8517)	99.8
S09-0039859	75,498,616	50,558,914	4,095,325,285	235 (1-1760)	99.7
S10-0029913	71,185,790	48,074,766	3,932,852,537	239 (1-1718)	99.7
S11-0004115	74,410,956	47,932,912	4,018,795,125	257 (1-3395)	99.8
S11-0009459	78,290,960	51,535,820	4,261,910,510	257 (1-9261)	99.8
S12-0025262	75,396,260	48,377,430	3,896,267,861	176 (1-2115)	99.7
S11-0010997	68,613,238	44,935,497	3,779,244,629	279 (1-3033)	99.7
S11-0037698	81,918,226	55,875,365	4,885,822,203	311 (1-2488)	99.8
S12-0002568	72,672,898	49,262,897	4,177,475,027	279 (1-1870)	99.8
S12-0046135	93,719,254	63,287,901	5,458,793,293	265 (1-17381)	99.8
S14-0014942	76,525,058	46,654,601	4,091,485,247	309 (1-8477)	99.7
S14-0040813	80,178,502	49,682,333	4,361,261,805	326 (1-2020)	99.8
S15-0029256	81,086,826	53,780,778	4,709,945,271	312 (1-3250)	99.7
S16-0002113	79,187,114	52,876,168	4,589,513,342	207 (1-6033)	99.7
S16-0005228	81,030,524	54,845,495	4,822,743,338	421 (1-2105)	99.7
S16-0022463	73,161,788	47,530,252	4,138,309,818	451 (1-2583)	99.8
S16-0037789	80,650,228	54,568,309	4,779,850,798	335 (1-4188)	99.7
S16-0044174	80,028,340	51,636,783	4,515,929,834	460 (1-3755)	99.8
S17-0005322	76,876,548	52,134,839	4,551,963,806	321 (1-10625)	99.8
S17-0022768	67,522,402	38,319,791	4,257,546,796	415 (1-2831)	99.7
S17-0037969	141,212,674	87,953,657	9,433,612,336	278 (1-2100)	99.7

(b)

Sample ID	Total number of sequenced reads	Total number of uniquely mapped reads	RNA integrity number (RIN)	Ratio of all reads aligned to rRNA regions to total uniquely mapped reads (rRNA rate)	Ratio of exon-mapped reads to total uniquely mapped reads (Expression Profile Efficiency)	Total number of detected transcripts with reads ≥1
S09-0039859	50,144,824	37,387,202	2.3	0.009	0.694	26,108
S10-0029913	45,081,107	34,621,201	2.5	0.010	0.838	26,314
S11-0009459	39,335,214	31,011,533	2.4	0.009	0.838	25,630
S11-0010997	52,176,373	41,226,744	2.4	0.010	0.857	28,952
S11-0037698	12,684,497	9,613,446	2.5	0.021	0.814	22,967
S12-0002568	51,004,620	42,736,108	2.4	0.013	0.851	28,615
S12-0011227	35,759,592	29,580,661	6.6	0.012	0.855	26,079
S12-0046135	63,872,018	53,203,090	2.3	0.013	0.878	32,662
S14-0014942	64,877,989	51,971,987	2.3	0.016	0.824	31,036
S14-0040813	58,294,856	47,906,467	1.5	0.012	0.854	33,584
S14-0046360	65,623,065	55,261,289	2.4	0.011	0.868	29,588
S15-0005814	51,614,677	39,485,327	2.7	0.018	0.747	27,271
S15-0011946	58,207,049	48,981,503	2.2	0.007	0.846	29,194
S15-0015504	22,526,451	12,683,070	2.3	0.003	0.532	25,185
S15-0024523	52,158,921	41,640,821	2.5	0.009	0.820	29,834
S15-0050616	42,991,580	35,354,771	2.2	0.006	0.878	25,116
S16-0002113	35,747,974	29,132,743	2.2	0.015	0.862	28,492
S16-0002170	57,077,741	39,287,199	2.4	0.015	0.856	29,600
S16-0005228	75,068,522	60,145,753	2.5	0.013	0.850	33,050
S16-0010218	80,410,808	65,615,778	2.5	0.009	0.856	30,382
S16-0010293	19,626,677	13,257,176	2.3	0.004	0.878	20,425
S16-0014112	61,145,044	50,408,603	2.5	0.010	0.851	29,876
S16-0015253	28,107,933	12,349,643	2.6	0.000	0.234	14,045
S16-0016629	18,747,472	14,557,503	2.5	0.001	0.844	19,868
S16-0022463	74,172,534	58,162,254	2.4	0.010	0.886	30,761
S16-0023700	76,925,302	59,655,545	2.5	0.015	0.774	32,934
S16-0027112	61,571,238	49,315,682	2.5	0.015	0.808	29,713
S16-0027501	51,737,833	43,575,324	2.5	0.015	0.849	28,766
S16-0032848	35,435,466	27,581,468	2.6	0.001	0.823	20,873
S16-0036144	75,608,724	57,751,695	2.5	0.009	0.797	28,614
S16-0037789	61,201,272	47,250,169	2.3	0.019	0.802	34,344
S16-0039501	53,056,667	40,927,560	2.5	0.011	0.815	30,745
S16-0040169	99,366,726	78,981,395	2.5	0.006	0.835	29,888
S16-0042813	40,913,280	27,917,630	2.5	0.006	0.789	23,404
S16-0044174	70,302,844	57,049,047	2.4	0.009	0.850	31,072
S16-0044542	55,753,637	46,576,840	2.5	0.008	0.853	31,253
S16-0046295	36,479,179	28,474,054	2.5	0.022	0.771	27,377
S16-0047073	60,317,943	49,692,609	2.6	0.013	0.822	30,582
S16-0047450	82,214,420	65,451,883	2.6	0.015	0.839	33,189
S16-0051220	67,254,892	51,108,858	2.6	0.007	0.813	29,708
S16-0052834	25,099,020	18,724,449	2.6	0.005	0.608	28,728
S17-0004535	71,472,849	56,741,165	2.5	0.006	0.864	32,230
S17-0005322	68,595,873	57,101,992	2.3	0.012	0.866	30,464
S17-0010308	44,706,829	34,836,092	2.5	0.010	0.799	28,664
S17-0013690N	69,598,811	52,797,332	2.6	0.010	0.789	27,323
S17-0013690	46,566,978	34,564,528	2.5	0.010	0.786	28,496
S17-0016300	53,740,656	43,042,725	2.6	0.006	0.868	26,702
S17-0022768	68,571,207	56,166,499	2.4	0.010	0.876	33,009
S17-0022784	28,451,910	21,417,156	5.6	0.008	0.789	23,793
S17-0037969	58,834,637	32,709,943	2.3	0.024	0.766	31,066
S17-0039675	29,436,652	14,981,778	1.1	0.000	0.258	18,055
S17-0047553	71,423,825	60,892,639	4.6	0.013	0.875	31,622
S17-0048117	39,415,298	29,954,921	2.5	0.009	0.774	29,637
S17-0052786	58,458,513	44,300,505	2.6	0.014	0.860	32,356
S17-0053053N	60,899,578	48,085,357	2.5	0.011	0.820	31,992
S17-0055533	23,237,916	18,125,121	1.9	0.005	0.808	24,070
S17-0056487	53,298,503	40,385,304	2.1	0.009	0.815	28,621
S18-0004302N	50,630,205	38,941,444	2.6	0.008	0.824	27,500
S18-0004302	53,761,036	43,403,164	2.6	0.008	0.856	32,415
S18-0013605	47,608,294	36,559,829	2.6	0.012	0.808	26,110
S18-0020718	46,938,944	38,963,900	2.3	0.018	0.841	31,141
S18-0023524	38,069,189	29,321,879	2.5	0.011	0.814	25,362
S18-0024830	39,715,745	30,283,445	2.5	0.016	0.776	31,057
S11-0004115	68,447,794	55,290,443	2.3	0.007	0.821	24,929
S15-0029256	29,390,484	20,067,836	-	0.001	0.464	21,804
S16-0050778	38,639,109	26,442,724	-	0.001	0.401	21,537
S09-0024194	57,019,857	46,060,291	-	0.009	0.822	25,707
S12-0025262	25,531,055	17,699,945	2.2	0.005	0.658	20,028
S17-0053053	52,758,494	28,712,363	2.4	0.004	0.609	25,935
