# Supplementary material for: Genetic and immune microenvironment characterization of HER2‐positive gastric cancer: Their association with response to trastuzumab‐based treatment
Source: Cancer Med. 2023 Mar 14;12(9):10371–84. doi: 10.1002/cam4.5769 (PMC10225221; doi:10.1002/cam4.5769)
Supplement: Supplementary file 4 — Table S3 [file CAM4-12-10371-s002.pdf]

Supplementary Table 3.

Antibody	Product	Antibody	Product
EGFR	Ready to use, 3C6, cat#790-2988, Ventana Medical Systems (Tucson, AZ, USA)	CD3	1:300, cat#A0452, DAKO
HER2	Ready to use, 4B5, cat#790-2991, Ventana Medical Systems	CD8	Redy to use, SP57, cat#790-4460, Ventana Medical Systems
HER3	1:100, SP71, cat#ab93739, Abcam	CD57	1:200, HNK-1/Leu-7, cat#ab187274, Abcam
CyclinA	1:1000, CY-A1, cat#C4710-2ML, Sigma-Aldrich (Darmstadt, Germany)	CD20	1:300, L26, cat#M0755, DAKO
CyclinD1	1:200, M-20, cat#sc-718, Santa-Cruz	CD68	1:100, PG-M1, cat#M0876, DAKO
CyclinD3	1:50, DCS-22, cat#MA5-12717, Invitrogen	CD163	1:500, OTI2G12, cat#ab156769, Abcam
CyclinE	1:100, HE12, cat#MA5-14336, Invitrogen	FOXP3	1:50, 236A/E7, cat#ab20034, Abcam
CDK6	1:1000, cat# sc-7961, Santa-Cruz BT, (California, USA)		
NRG1	1:2000, cat#ab53104, Abcam		
PLK1	1:1000, cat #4513, Cell signaling, (Beverly, MA, USA)		
P53	1:1000, DO-7, cat#M7001, DAKO		
P27	1:300, cat#E2604, Spring Bioscience		
P21	1:200, 187, cat#sc-817, Santa-Cruz		
RB	1:100, G3-245, cat#554136, BD Biosciences		
Ki-67	1:100, MIB-1, cat#M7240, DAKO		
PD-L1	1:30, 22C3, cat#M3653, DAKO		
