# Supplementary material for: Genetic and immune microenvironment characterization of HER2‐positive gastric cancer: Their association with response to trastuzumab‐based treatment
Source: Cancer Med. 2023 Mar 14;12(9):10371–84. doi: 10.1002/cam4.5769 (PMC10225221; doi:10.1002/cam4.5769)
Supplement: Supplementary file 5 — Table S4 [file CAM4-12-10371-s005.pdf]

Supplementary Table 4.

KEGG pathways	Size	ES	NES	NOM p-val	FDR q-val	FWER p-val
**Up regulated in Group 1 than Group 2**						
RIBOSOME	86	0.63	2.22	0.000	0.000	0.000
SPLICEOSOME	125	0.56	2.05	0.000	0.000	0.001
AMINOACYL TRNA BIOSYNTHESIS	41	0.66	2.00	0.000	0.000	0.001
CELL CYCLE	124	0.55	1.98	0.000	0.000	0.001
RNA DEGRADATION	54	0.63	1.98	0.000	0.000	0.001
PROTEASOME	44	0.63	1.96	0.000	0.000	0.002
HUNTINGTONS DISEASE	160	0.51	1.92	0.000	0.000	0.004
PARKINSONS DISEASE	101	0.54	1.89	0.000	0.001	0.009
DNA REPLICATION	36	0.62	1.81	0.000	0.003	0.036
NUCLEOTIDE_EXCISION_REPAIR	44	0.58	1.78	0.001	0.004	0.054
TERPENOID BACKBONE BIOSYNTHESIS	15	0.72	1.74	0.003	0.008	0.094
UBIQUITIN MEDIATED PROTEOLYSIS	133	0.47	1.71	0.000	0.011	0.138
BASAL TRASCRIPTION FACTORS	35	0.58	1.70	0.001	0.012	0.163
OXIDATIVE PHOSPHORYLATION	101	0.48	1.68	0.000	0.015	0.208
OOCYTE MEIOSIS	112	0.46	1.66	0.000	0.018	0.257
PYRIMIDINE METABOLISM	98	0.48	1.66	0.001	0.018	0.265
CITRATE CYCLE TCA CYCLE	30	0.58	1.64	0.008	0.021	0.327
PROTEIN EXPORT	23	0.58	1.60	0.009	0.031	0.477
MISMATCH REPAIR	23	0.59	1.59	0.008	0.031	0.501
PROGESTERONE MEDIATED OOCYTE MATURATION	85	0.43	1.49	0.005	0.069	0.859
**Up regulated in Group 2 than Group 1**						
HEMATOPOIETIC CELL LINEAGE	85	-0.52	-1.99	0.000	0.005	0.005
ALLOGRAFT REJECTION	35	-0.61	-1.93	0.000	0.005	0.010
ARRHYTHMOGENIC RIGHT VENTRICULAR CARDIOMYOPATHY ARVC	74	-0.51	-1.89	0.003	0.007	0.020
CELL ADHESION MOLECULES CAMS	131	-0.46	-1.85	0.000	0.010	0.037

DILATED CARDIOMYOPATHY	90	-0.46	-1.76	0.000	0.023	0.092
NEUROACTIVE LIGAND RECEPTOR INTERACTION	271	-0.40	-1.72	0.000	0.030	0.149
AUTOIMMUNE THYROID DISEASE	50	-0.50	-1.68	0.000	0.034	0.191
VASCULAR SMOOTH MUSCLE CONTRACTION	113	-0.42	-1.68	0.000	0.032	0.203
GRAFT VERSUS HOST DISEASE	37	-0.51	-1.67	0.002	0.030	0.216
HYPERTROPHIC CARDIOMYOPATHY HCM	83	-0.44	-1.67	0.000	0.028	0.223
ASTHMA	28	-0.56	-1.67	0.002	0.025	0.223
LEISHMANIA INFECTION	70	-0.46	-1.66	0.000	0.024	0.236
TYPE 1 DIABETES MELLITUS	41	-0.50	-1.66	0.000	0.024	0.249
PRIMARY IMMUNODEFICIENCY	35	-0.51	-1.64	0.008	0.026	0.279
INTESTINAL IMMUNE NETWORK FOR IGA PRODUCTS	45	-0.49	-1.63	0.005	0.026	0.298
CALCIUM SIGNALING PATHWAY	177	-0.39	-1.63	0.000	0.025	0.309
CYTOKINE CYTOKINE RECEPTOR INTERACTION	262	-0.36	-1.58	0.000	0.036	0.426
FOCAL ADHESION	199	-0.37	-1.56	0.000	0.040	0.479
ECM RECEPTOR INTERACTION	84	-0.40	-1.51	0.003	0.053	0.620
**Up regulated in pre-treatment than post-treatment**						
RIBOSOME	86	-0.58	-1.70	0.000	0.069	0.080
DNA_REPLICATION	36	-0.65	-1.65	0.002	0.080	0.170
ARRHYTHMOGENIC RIGHT VENTRICULAR CARDIOMYOPATHY ARVC	74	-0.56	-1.62	0.002	0.086	0.268
**Up regulated in post-treatment than pre-treatment**						
ALLOGRAFT REJECTION	35	0.74	2.10	0.000	0.000	0.000
ASTHMA	28	0.76	2.02	0.000	0.000	0.000
GRAFT VERSUS HOST DISEASE	37	0.69	1.99	0.000	0.000	0.001
COMPLEMENT AND COAGULATION CASCADES	69	0.62	1.98	0.000	0.000	0.001
AUTOIMMUNE THYROID DISEASE	50	0.65	1.97	0.000	0.000	0.001

RETINOL METABOLISM	64	0.61	1.88	0.000	0.002	0.012
ANTIGEN PROCESSING AND PRESENTATION	81	0.56	1.82	0.000	0.005	0.029
GLYCOSAMINOGLYCAN BIOSYNTHESIS CHONDROITIN SULFATE	22	0.68	1.73	0.005	0.017	0.095
INTESTINAL IMMUNE NETWORK FOR IGA PRODUCTS	45	0.57	1.66	0.003	0.035	0.209
TYPE 1 DIABETES MELLITUS	41	0.56	1.61	0.003	0.050	0.308
LEISHMANIA INFECTION	70	0.50	1.56	0.000	0.065	0.441

Size, gene set size; ES, enrichment score; NES, normalized enrichment score; NOM p-val, normalized p-value; FDR q-val, false discovery rate q-value; FWER p-val, adjusted p-value of familywise-error rate.
