# Supplementary figures and images for: Genetic and immune microenvironment characterization of HER2‐positive gastric cancer: Their association with response to trastuzumab‐based treatment
Source: Cancer Med. 2023 Mar 14;12(9):10371–84. doi: 10.1002/cam4.5769 (PMC10225221; doi:10.1002/cam4.5769)

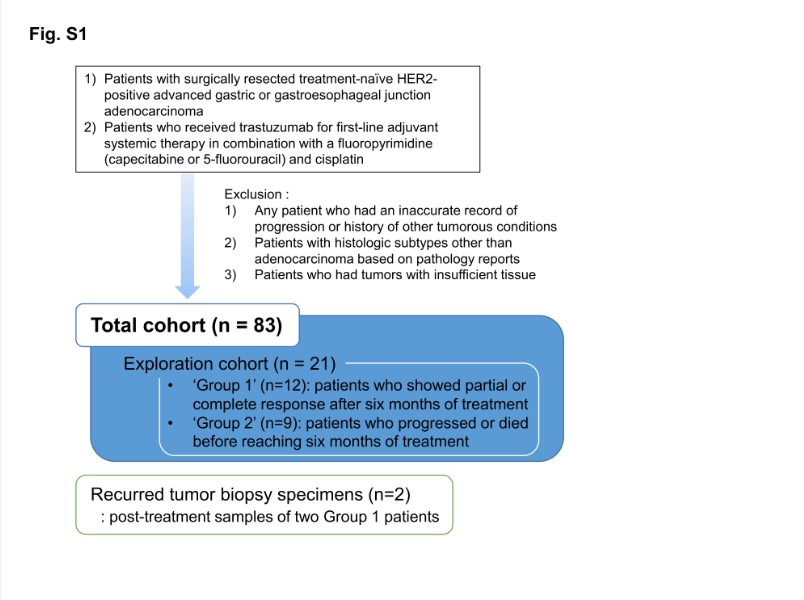

Supplement: Supplementary file 6 — Figure S1 [file CAM4-12-10371-s003.jpg]

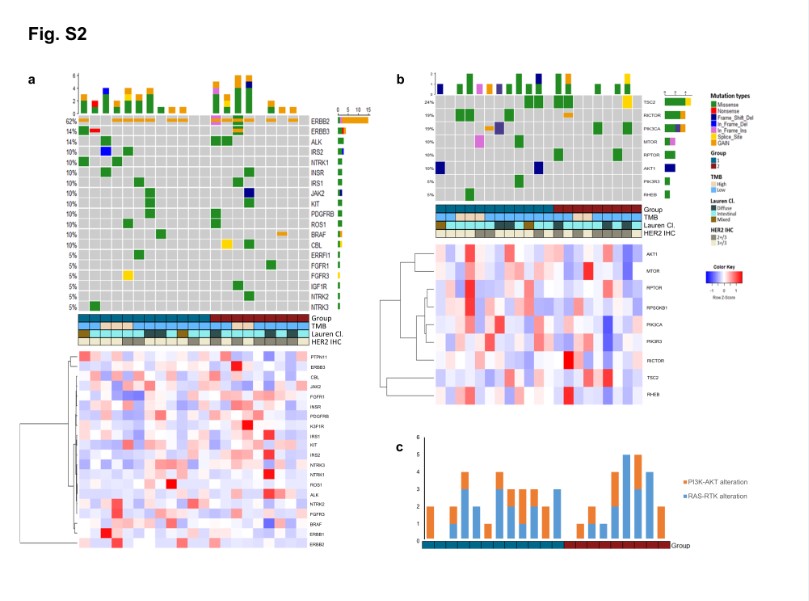

Supplement: Supplementary file 7 — Figure S2 [file CAM4-12-10371-s010.jpg]

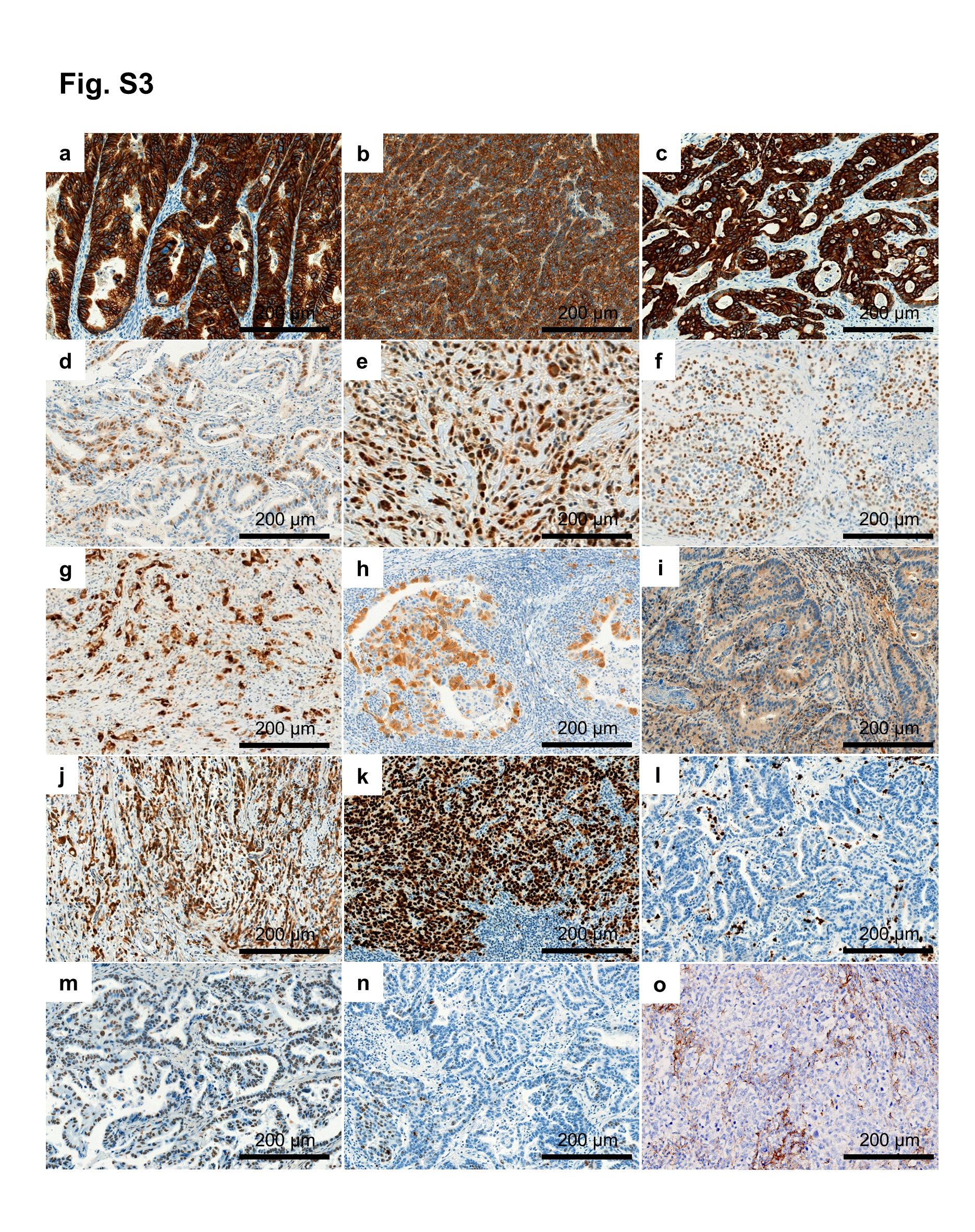

Supplement: Supplementary file 8 — Figure S3 [file CAM4-12-10371-s008.tif]

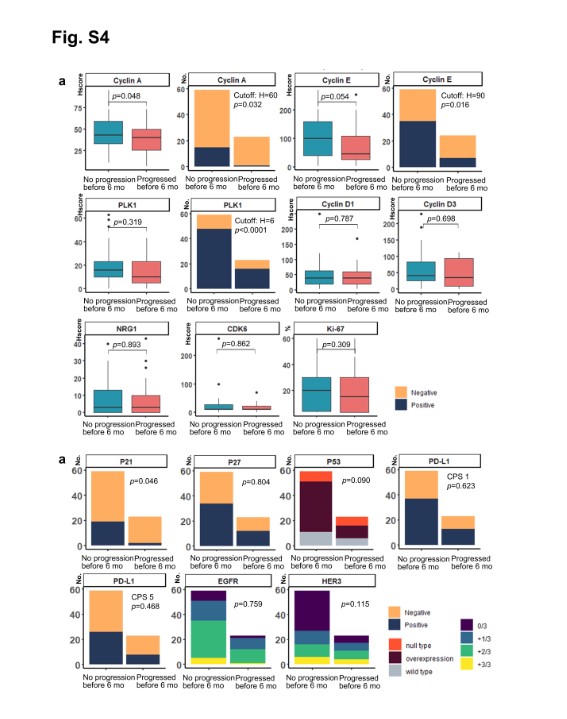

Supplement: Supplementary file 9 — Figure S4 [file CAM4-12-10371-s009.jpg]

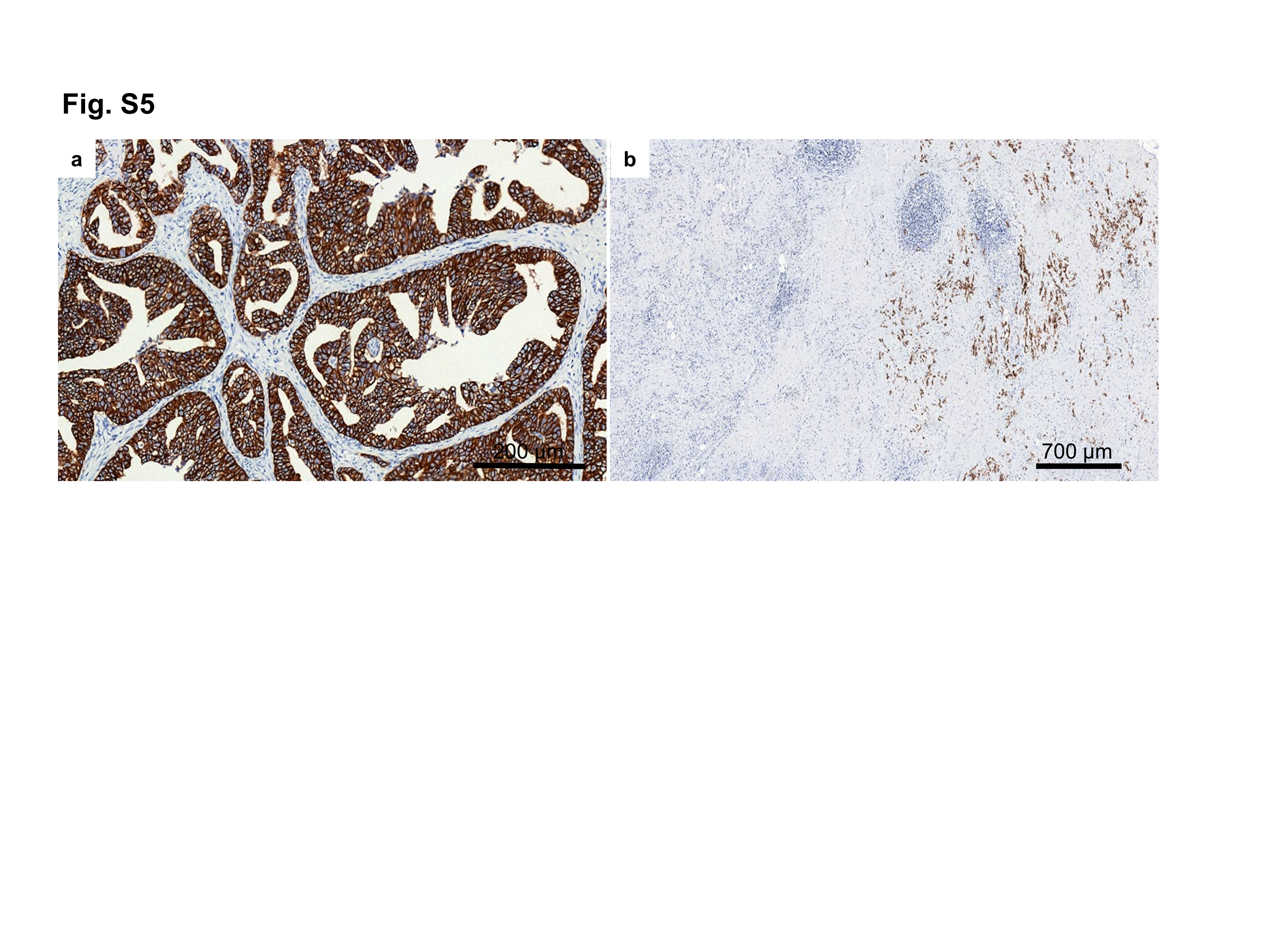

Supplement: Supplementary file 10 — Figure S5 [file CAM4-12-10371-s007.tif]
